# Maternal and Neonatal Outcomes Following Antibiotic Prophylaxis in Term Prelabor Rupture of Membranes (PROM): Evidence From a Systematic Review

**DOI:** 10.7759/cureus.99680

**Published:** 2025-12-20

**Authors:** Mohammedelfateh Adam, Aya Eltom Hajo Elsheikh, Ryan Osman Alhessen Saidahmed, Rawan Awad Amer Ahmed, Jihan Ahmed Osman Duffuaa

**Affiliations:** 1 Obstetrics and Gynecology, Cork University Maternity Hospital, Cork, IRL; 2 Obstetrics and Gynecology, Sudanese Medical Specialisation Board, Khartoum, SDN; 3 Obstetrics and Gynecology, Sabt Al Alaya General Hospital, Sabt Al Alayah, SAU; 4 Anatomical Sciences, St George's University, St George's, GRD; 5 Obstetrics and Gynecology, University of Khartoum, Khartoum, SDN

**Keywords:** antibiotic prophylaxis, chorioamnionitis, maternal outcomes, neonatal outcomes, neonatal sepsis, systematic review, term premature rupture of membranes

## Abstract

Term prelabor rupture of membranes (PROM) is a common obstetric event associated with an increased risk of maternal and neonatal infectious morbidity. The use of antibiotic prophylaxis to prevent these complications is widespread, yet its efficacy remains debated due to conflicting evidence and concerns regarding antimicrobial resistance. This systematic review aimed to synthesize the existing evidence on the impact of antibiotic prophylaxis on maternal and neonatal outcomes in term PROM. A systematic literature search was conducted following Preferred Reporting Items for Systematic Reviews and Meta-Analyses (PRISMA) guidelines across PubMed/MEDLINE, Web of Science, Embase, and ClinicalTrials.gov from inception. Studies including pregnant women with term PROM (≥37 weeks) comparing antibiotic prophylaxis to placebo, no treatment, or different timing strategies were eligible. Two reviewers independently screened studies, extracted data, and assessed the risk of bias using the Cochrane Risk of Bias 2 (RoB 2) tool for randomized controlled trials (RCTs) and the Risk of Bias in Non-randomized Studies - of Interventions (ROBINS-I) tool for non-randomized studies. A narrative synthesis was performed due to significant clinical and methodological heterogeneity. Six studies (four RCTs and two retrospective cohorts) were included. The findings were inconsistent. Three studies reported a significant reduction in maternal infections (chorioamnionitis, endometritis) with antibiotic prophylaxis, while two large studies found no significant benefit. For neonatal outcomes, two studies reported a significant reduction in neonatal sepsis, whereas three others found no statistically significant difference. Evidence regarding the optimal timing of antibiotic administration was also conflicting, with one high-quality study finding no benefit for administration within 6-12 hours versus later, while others suggested earlier administration was associated with better outcomes. The overall risk of bias was low for the majority of the included studies. The evidence supporting the routine use of antibiotic prophylaxis for all women with term PROM was inconclusive. While a reduction in maternal infection was observed in some studies, this benefit was not universal, and the effect on neonatal sepsis remains uncertain. A targeted approach, considering individual risk factors such as prolonged membrane rupture or Group B Streptococcus colonization, may be more appropriate than universal prophylaxis. Further high-quality research is needed to define the optimal timing and candidate population for this intervention.

## Introduction and background

Premature rupture of membranes (PROM) is defined as the spontaneous rupture of the fetal membranes before the onset of labor and occurs in approximately 8-10% of all pregnancies [1]. When membrane rupture occurs at or beyond 37 completed weeks of gestation, it is classified as term PROM, in contrast to preterm PROM, which occurs before 37 weeks. A key concept in PROM management is the latency period, referring to the interval between membrane rupture and delivery. Term PROM poses important clinical challenges, particularly regarding the optimal timing of delivery and the role of antibiotic prophylaxis, both of which remain areas of ongoing debate [2]. The principal clinical concern is the increased susceptibility to maternal and neonatal infections resulting from prolonged exposure of the uterine cavity to ascending vaginal microorganisms, leading to complications such as chorioamnionitis, postpartum endometritis, and neonatal sepsis [3]. Although the absolute risk varies, infectious morbidity has been reported in a clinically meaningful proportion of term PROM cases, underscoring its relevance to obstetric practice.

Antibiotic prophylaxis has been proposed as a preventive strategy to reduce infectious complications associated with term PROM, particularly during prolonged latency or in the presence of suspected infection [4]. Several professional guidelines advocate selective rather than universal antibiotic use, generally recommending antibiotics when clinical signs of infection are present or when the latency period exceeds predefined thresholds [5]. However, recommendations differ across guidelines, reflecting uncertainty in the evidence base. Moreover, routine antibiotic administration is not without risk, as unnecessary exposure may contribute to antimicrobial resistance, disruption of maternal and neonatal microbiota, and adverse drug reactions. Consequently, identifying patient subgroups most likely to benefit from prophylaxis while minimizing potential harms remains a critical clinical priority [6].

Despite extensive investigation over recent decades, evidence regarding the effectiveness of antibiotic prophylaxis in term PROM remains inconsistent [1,2,7]. While some studies suggest a reduction in maternal infectious morbidity or neonatal sepsis, others report little to no benefit, particularly among women with shorter latency periods [1]. Interpretation of these findings is further complicated by heterogeneity in study design, antibiotic regimens, timing of administration, and definitions of infectious outcomes [7]. These methodological variations limit the generalizability of individual studies and contribute to uncertainty in clinical decision-making.

Accordingly, a clear gap persists in the literature regarding whether routine or selective antibiotic prophylaxis meaningfully improves maternal and neonatal outcomes in term PROM and under what clinical circumstances such intervention is justified. This systematic review aimed to synthesize and critically appraise the available evidence on maternal and neonatal outcomes following antibiotic prophylaxis in term PROM. By analyzing studies published over the past decade, this review sought to clarify the benefits and potential risks of antibiotic use, address inconsistencies in existing data, and support more informed, evidence-based management strategies for women presenting with term PROM.

## Review

Methodology

Protocol and Registration

This systematic review was conducted in accordance with the Preferred Reporting Items for Systematic Reviews and Meta-Analyses (PRISMA) 2020 guidelines to ensure transparency and reproducibility [8]. The protocol was developed prior to the commencement of the review process, outlining the objectives, eligibility criteria, and methodological approach. Although the review was not prospectively registered in the International Prospective Register of Systematic Reviews (PROSPERO), all steps were carried out according to PRISMA standards to maintain methodological rigor and consistency.

Information Sources and Search Strategy

A comprehensive literature search was performed across four major electronic databases: PubMed/MEDLINE, Web of Science, Embase, and ClinicalTrials.gov. The search strategy was designed to identify all relevant studies assessing the impact of antibiotic prophylaxis on maternal and neonatal outcomes in women with term PROM. A combination of Medical Subject Headings (MeSH) terms and free-text keywords such as “term premature rupture of membranes”, “antibiotic prophylaxis”, “maternal outcomes”, and “neonatal outcomes” was used. Boolean operators (AND, OR) and truncation symbols were applied where appropriate to broaden or narrow the search results. To ensure comprehensive coverage, no restriction was placed on the publication date or language, thereby allowing the inclusion of both recent and older studies relevant to the research question. Reference lists of eligible studies and related reviews were also screened manually to identify additional relevant articles. The detailed search strategy for each database is provided in Appendix A.

Eligibility Criteria 

Eligibility criteria were formulated using the Population, Intervention, Comparison, Outcomes, and Study Design (PICOS) framework. The population (P) included pregnant women with term PROM (≥37 weeks of gestation). The intervention (I) consisted of antibiotic prophylaxis administered either before or during labor following membrane rupture. The comparator (C) included either placebo, no antibiotic prophylaxis, or different antibiotic regimens. The outcomes (O) assessed were both maternal outcomes (such as chorioamnionitis, endometritis, and postpartum infections) and neonatal outcomes (including early-onset sepsis, neonatal intensive care unit admission, and perinatal mortality). The study designs (S) considered included randomized controlled trials (RCTs) and observational studies (cohort and case-control designs). Studies focusing on preterm PROM, animal experiments, reviews, case reports, editorials, and conference abstracts were excluded.

Study Selection and Data Management

All retrieved records from the databases were imported into EndNote X9 software (Clarivate, London, UK), where duplicate entries were identified and removed automatically and manually. Two independent reviewers screened the titles and abstracts to identify potentially relevant studies. The full texts of the selected articles were then assessed against the inclusion and exclusion criteria. Any disagreements between the reviewers were resolved through discussion or consultation with a third reviewer to achieve consensus. Data from the included studies were extracted using a structured data extraction form, capturing details such as author, year of publication, country, study design, sample size, population characteristics, type and timing of antibiotic intervention, comparator, follow-up duration, and reported maternal and neonatal outcomes.

Assessment of Risk of Bias in Included Studies

The methodological quality and risk of bias of the included studies were independently assessed by two reviewers. For RCTs, the Cochrane Risk of Bias 2 (RoB 2) tool [9] was employed, evaluating domains such as randomization process, deviations from intended interventions, missing outcome data, measurement of outcomes, and selection of reported results. For non-randomized studies, the Risk of Bias in Non-randomized Studies - of Interventions (ROBINS-I) tool [10] was used, assessing potential confounding, participant selection, intervention classification, deviations from intended interventions, missing data, outcome measurement, and result selection. Any discrepancies were resolved by consensus to ensure accuracy and objectivity.

Data Synthesis and Rationale for Not Performing Meta-Analysis

Given the considerable heterogeneity among the included studies in terms of antibiotic regimens (types, doses, timing, and duration), study designs, outcome definitions, and reporting standards, it was deemed inappropriate to perform a meta-analysis. Pooling such heterogeneous data could lead to misleading or unreliable effect estimates. Instead, a narrative synthesis was conducted to summarize and interpret the evidence qualitatively. The findings were organized thematically to highlight trends in maternal and neonatal outcomes, as well as to identify gaps in the current literature that warrant further investigation.

Results

Study Selection Process

**Figure 1:**
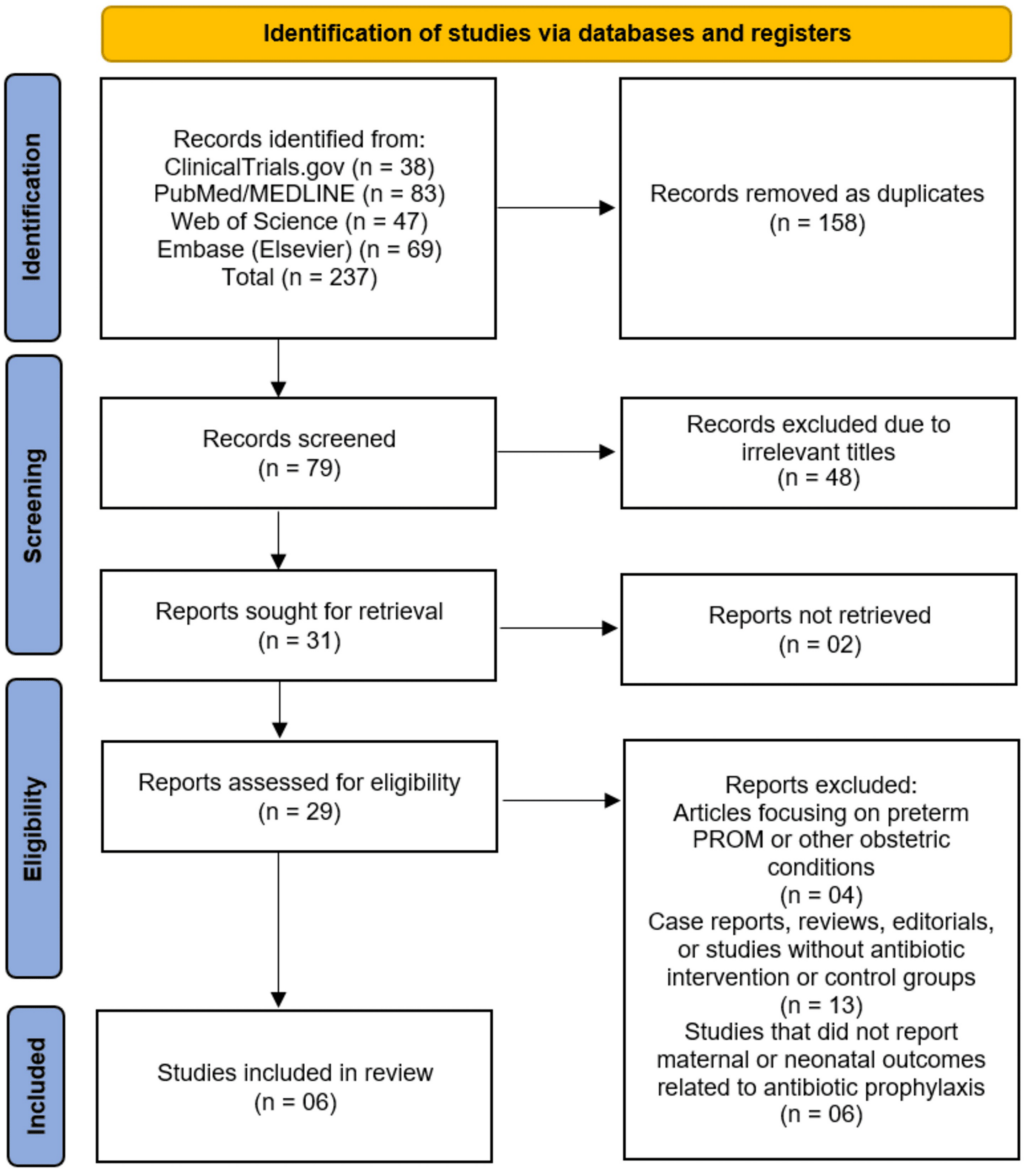
Studies selection process illustrated on PRISMA flowchart PRISMA: Preferred Reporting Items for Systematic Reviews and Meta-Analyses; PROM: prelabor rupture of membranes

The systematic search across four databases and registers (PubMed/MEDLINE, Web of Science, Embase, and ClinicalTrials.gov) initially identified 237 records. After the removal of 158 duplicate records, a total of 79 unique studies were screened based on their titles and abstracts. This screening phase led to the exclusion of 48 records that were deemed irrelevant to the review's focus. The full texts of the remaining 31 articles were sought for retrieval, of which 29 were successfully obtained. A detailed eligibility assessment of these 29 reports resulted in the exclusion of 23 studies. The reasons for exclusion were: a focus on preterm PROM or other obstetric conditions (n=4); being a case report, review, editorial, or lacking a relevant intervention or control group (n=13); and not reporting on the specific maternal or neonatal outcomes related to antibiotic prophylaxis (n=6). Ultimately, this rigorous selection process yielded six studies that met all the pre-defined inclusion criteria for the systematic review (Figure 1) [11-16].

Description of Included Studies

A total of six studies [11-16] were included in this systematic review to evaluate the efficacy of antibiotic prophylaxis on maternal and neonatal outcomes in cases of term PROM. The characteristics of these studies are summarized in Table 1. The included studies were published between 1998 and 2024 and were conducted in diverse geographical locations, including China [11], Portugal [12], Bosnia and Herzegovina [13], Egypt [14], Chile [15], and Spain [16]. The sample sizes varied, with maternal cohorts ranging from 105 [15] to 5,353 [11].

**Table 1:** Characteristics of included studies RCT: randomized controlled trial; IV: intravenous; PROM: prelabor rupture of membranes

Author (year)	Country	Study design	Sample size (maternal / neonatal)	Intervention	Comparator	Follow-up duration
Dan et al. (2024) [11]	China	Retrospective cohort study using propensity‑score matching	5353	Antibiotic prophylaxis within 6 hours or 12 hours of PROM	Antibiotic prophylaxis after 6 hours or 12 hours of PROM	42 days (for maternal puerperal infection)
Passos et al. (2012) [12]	Portugal	Randomized controlled nonblinded trial	161 / 161	Prophylactic antibiotics	No treatment / usual care	Until delivery and early neonatal period
Barišić et al. (2017) [13]	Bosnia and Herzegovina	Retrospective cohort study	144 / 144	Ampicillin within 6 hours of PROM	Ampicillin after 6 hours of PROM	Until hospital discharge of newborns
Nabhan et al. (2014) [14]	Egypt (Cairo)	RCT	1640 / not explicitly reported	Single dose of prophylactic IV antibiotics	Placebo	From admission to early neonatal period
Ovalle et al. (1998) [15]	Chile	RCT	105 (55 maternal in intervention, 50 maternal in placebo)	IV cefuroxime + clindamycin	Placebo	Until hospital discharge
Cararach et al. (1998) [16]	Spain	Prospective, randomized, multicenter study	733 / not explicitly reported (assumed same as maternal)	Antibiotic administration after PROM	No antibiotics (control)	Until delivery and immediate neonatal period

The designs of the included studies were a mix of RCTs [12,14-16] and retrospective cohort studies [11,13]. Among the RCTs, one was a non-blinded trial [12], while others utilized a placebo control [14,15]. The retrospective studies employed propensity-score matching [11] or comparative cohort analysis [3] to enhance the validity of their comparisons. The interventions primarily consisted of various antibiotic regimens (e.g., ampicillin, cefuroxime, clindamycin) administered prophylactically after PROM, compared to either placebo [14,15] or no treatment/usual care [12,16]. One study specifically investigated the timing of antibiotic administration, comparing initiation within six or 12 hours versus after these time thresholds [11]. The follow-up duration for outcomes typically extended until maternal hospital discharge or the early neonatal period.

Maternal Outcomes

The impact of antibiotic prophylaxis on maternal infectious morbidity was a key outcome across all studies, though the findings were not entirely consistent. Three studies reported a statistically significant reduction in maternal infections with the use of antibiotics. Passos et al. [12] found that antibiotic prophylaxis significantly lowered the rate of maternal infection (chorioamnionitis and endometritis) compared to the control group (2.6% vs. 13.2%; risk ratio (RR) 0.89; 95% CI 0.81-0.98). Similarly, Ovalle et al. [15] observed a marked reduction in clinical chorioamnionitis and puerperal endometritis in the antibiotic group (1.8% vs. 16%). Barišić et al. [13] also reported benefits, noting a lower incidence of chorioamnionitis (3.47% vs. 9.72%) and a lower median C-reactive protein level when antibiotics were given within six hours of PROM.

In contrast, two studies found no significant benefit for maternal outcomes. Dan et al. [11] concluded that the timing of antibiotic prophylaxis (within or after 6-12 hours of PROM) did not significantly affect the rate of puerperal infection. Nabhan et al. [14] also reported no significant difference in maternal infection-related morbidity between the antibiotic and placebo groups. Cararach et al. [16] reported a reduction in endometritis with antibiotics, but this finding was not statistically significant.

Neonatal Outcomes

The evidence regarding neonatal outcomes following antibiotic prophylaxis was similarly mixed. Two studies demonstrated a significant beneficial effect on neonatal infections. Cararach et al. [16] reported a statistically significant reduction in neonatal sepsis in the antibiotic group (one vs. seven cases, p = 0.007). Barišić et al. [13] also found that early antibiotic administration was associated with a lower frequency of neonatal infection (15.4% vs. 45.3%) and a shorter neonatal hospital stay.

However, three other studies found no statistically significant effect of antibiotic prophylaxis on neonatal infectious outcomes. Passos et al. [12] noted a lower numerical rate of neonatal infection in the antibiotic group (3.8% vs. 6.0%), but this difference was not significant (p = 0.375). Nabhan et al. [14] found a slightly higher, though not significant, rate of early-onset neonatal sepsis in the antibiotic group compared to placebo (4.1% vs. 2.9%; RR 1.42, 95% CI 0.85-2.37). Dan et al. [11] also reported no significant difference in the rate of neonatal sepsis or total neonatal infection based on the timing of antibiotic administration. Ovalle et al. [15] did not report significant differences in neonatal outcomes between their study groups. A summary of these maternal and neonatal outcomes is provided in Table 2.

**Table 2:** Maternal and neonatal outcomes following antibiotic prophylaxis PROM: prelabor rupture of membranes; RR: risk ratio

Author (year)	Maternal outcomes	Neonatal outcomes	Key findings
Dan et al. (2024) [11]	Puerperal infection: 4.6% vs. 4.3% (≤6 hours vs. >6 hours), 2.9% vs. 4.6% (≤12 hours vs. >12 hours); no significant difference	Neonatal sepsis and total neonatal infection: no significant difference between early and late antibiotic groups	No statistical difference in maternal or neonatal infections whether antibiotic prophylaxis was initiated within 6-12 hours or after 6-12 hours of PROM. Cost‑minimization analysis showed similar direct medical costs between groups
Passos et al. (2012) [12]	Maternal infection (chorioamnionitis, endometritis) significantly lower with antibiotics (2.6% vs. 13.2%; RR 0.89; 95% CI 0.81-0.98; p = 0.013). All infections occurred in women with >12 hours of PROM	Neonatal infection lower in antibiotic group (3.8% vs. 6.0%), but not statistically significant (p = 0.375)	Antibiotic prophylaxis in term PROM significantly reduced maternal infection; effect on neonatal infection was not statistically significant
Barišić et al. (2017) [13]	Lower median C-reactive protein (3.0 ± 2.9 mg/L vs. 6.1 ± 7.3 mg/L), shorter time between PROM and delivery (p < 0.001), lower incidence of chorioamnionitis (3.47% vs. 9.72%)	Shorter hospital stay (4.13 vs. 4.94 days; p = 0.023), lower frequency of neonatal infection (15.4% vs. 45.3%)	Antibiotics given within 6 hours of PROM associated with improved maternal and neonatal outcomes; delayed administration linked to higher neonatal infection and maternal chorioamnionitis
Nabhan et al. (2014) [14]	No significant difference in maternal infection-related morbidity between antibiotic and placebo groups	Early-onset neonatal sepsis: 4.1% (antibiotics) vs. 2.9% (placebo); RR 1.42 (95% CI 0.85-2.37)	Routine prophylactic antibiotics in term PROM (≥36 weeks) do not reduce maternal or neonatal infection-related morbidity
Ovalle et al.(1998) [15]	Lower incidence of maternal infection-related morbidity (clinical chorioamnionitis and puerperal endometritis): 1.8% in antibiotic group vs. 16% in placebo group (p < 0.05)	No significant differences reported between groups	Antibiotic administration in term PROM significantly reduces maternal infection-related morbidity but does not affect other maternal or neonatal outcomes
Cararach et al. (1998) [16]	Chorioamnionitis: reduced (not statistically significant). Endometritis: reduced	Neonatal sepsis: significantly lower in antibiotic group (1 vs. 7 cases, p = 0.007)	Prophylactic antibiotics in term PROM (≥36 weeks) significantly reduce neonatal sepsis and likely reduce maternal endometritis, though maternal outcome differences were not statistically significant

Timing of Antibiotic Administration

One study specifically investigated the critical window for antibiotic administration. Dan et al. [11] focused on the timing of prophylaxis and found no statistical difference in maternal or neonatal infection rates whether antibiotics were initiated within 6-12 hours or after 6-12 hours of PROM. This suggests that, within this timeframe, the timing may not be a decisive factor for infection outcomes. Conversely, the findings from Barišić et al. [13] and Passos et al. [12] imply that earlier administration may be more beneficial, with the latter noting that all maternal infections in their trial occurred in women with more than 12 hours of PROM [12].

Risk of Bias Assessment

The methodological quality of the included studies was assessed using the Cochrane RoB 2 tool for RCTs [12,14-16] and the ROBINS-I tool for non-randomized cohort studies [11,13]. The overall risk of bias was low for the majority of studies. Among the RCTs, three were judged to have a low risk of bias [12,14,16], indicating confidence in their results, while one RCT was rated as high risk due to concerns in the randomization process and a lack of blinding [15]. For the non-randomized studies, both retrospective cohort analyses were assessed as having a low risk of bias [11,13], suggesting that the use of robust methodologies like propensity-score matching [11] and careful cohort selection [13] effectively mitigated potential biases from confounding and other non-randomized design elements. Consequently, the body of evidence synthesized in this review is predominantly comprised of studies with a low risk of bias, strengthening the reliability of the findings (Tables 3-4).

**Table 3:** Risk of bias assessment for RCTs using the Cochrane RoB 2 tool RoB 2: Risk of Bias 2; RCT: randomized controlled trial

Author (year)	Bias arising from the randomization process	Bias due to deviations from intended interventions	Bias due to missing outcome data	Bias in measurement of the outcome	Bias in selection of the reported result	Overall risk of bias
Passos et al. (2012) [12]	Low	Low	Low	Low	Low	Low
Nabhan et al. (2014) [14]	Low	Low	Low	Low	Low	Low
Ovalle et al. (1998) [15]	Some concerns	High	Low	Low	Some concerns	High
Cararach et al. (1998) [16]	Low	Low	Low	Low	Low	Low

**Table 4:** Risk of bias assessment for cohort studies using the ROBINS-I tool ROBINS-I: Risk of Bias in Non-randomized Studies - of Interventions

Author (year)	Bias due to confounding	Bias in selection of participants	Bias in classification of interventions	Bias due to deviations from intended interventions	Bias due to missing data	Bias in measurement of outcomes	Bias in selection of the reported result	Overall risk of bias
Dan et al. (2024) [11]	Low	Low	Low	Low	Low	Low	Low	Low
Barišić et al. (2017) [13]	Low	Low	Low	Low	Low	Low	Low	Low

Discussion

This systematic review synthesized evidence from six studies to evaluate the efficacy of antibiotic prophylaxis on maternal and neonatal outcomes in cases of term PROM. The collective findings present a complex and nuanced picture, indicating that while antibiotic prophylaxis can be beneficial in specific contexts, its universal application for term PROM is not unequivocally supported. The evidence suggests that the decision to administer antibiotics may hinge on specific clinical circumstances, particularly the timing of administration and the outcomes deemed most critical, rather than constituting a one-size-fits-all intervention.

The most compelling evidence for the use of antibiotic prophylaxis emerges in the context of reducing maternal infectious morbidity. Three studies in this review demonstrated a statistically significant reduction in maternal infections. Passos et al. [12] reported a substantial decrease in chorioamnionitis and endometritis, while Ovalle et al. [15] and Barišić et al. [13] observed similar protective effects. This aligns with the biological plausibility that antibiotics can prevent ascending genital tract infections in the vulnerable period between membrane rupture and delivery [17]. The findings from Barišić et al. [13] and Passos et al. [12] further suggest that the risk of maternal infection is not static but increases with the duration of membrane rupture, implying that any benefit of prophylaxis might be most pronounced in scenarios where delivery is delayed. This is consistent with the rationale underpinning the use of antibiotics in preterm PROM, where prolonged latency is common and the risk of infection is well-established. The significant reduction in maternal infection shown in these studies provides a strong argument for considering antibiotic prophylaxis, particularly for women who are not in active labor or for whom a delay in delivery is anticipated.

However, this favorable view is tempered by studies that found no significant benefit for maternal outcomes. Both Dan et al. [11] and Nabhan et al. [14] reported no statistically significant difference in maternal infection rates between their comparison groups. The divergence in findings could be attributed to several factors. The study by Nabhan et al. [14] was a large, placebo-controlled RCT, a design considered the gold standard for minimizing bias, and its null finding carries considerable weight. It is possible that in their population, the baseline risk of infection was lower, or that standard obstetric management, including a more active approach to induction of labor, minimized the window of vulnerability that antibiotics aim to address. The study by Dan et al. [11], which focused specifically on the timing of antibiotics rather than their use versus non-use, further complicates the narrative. Their finding that initiating prophylaxis within six or 12 hours was not superior to later administration challenges the intuitive clinical impulse for early intervention and suggests that the critical period for preventing infection may be longer than previously assumed, or that other factors, such as the total duration of rupture, are more consequential than the timing of the first antibiotic dose.

When considering neonatal outcomes, the evidence becomes even more equivocal and raises important considerations for clinical practice. The significant reduction in neonatal sepsis reported by Cararach et al. [16] and the lower frequency of neonatal infection found by Barišić et al. [13] offer a compelling rationale for prophylaxis, as the prevention of serious neonatal infection is a paramount concern. However, these encouraging findings are counterbalanced by the results of other high-quality studies. Passos et al. [12], Nabhan et al. [14], and Dan et al. [11] all failed to find a statistically significant benefit for neonatal infectious outcomes. Notably, the large trial by Nabhan et al. [14] even reported a non-significant trend toward a higher rate of early-onset neonatal sepsis in the antibiotic group, a finding that, while not conclusive, underscores the potential for unintended consequences. The administration of antibiotics to the mother disrupts the maternal and, subsequently, the neonatal microbiome. This disruption has been theorized to potentially increase the risk of other neonatal conditions, such as necrotizing enterocolitis (though more common in preterm infants) or late-onset infections with resistant organisms, a concern highlighted in studies outside this review. Therefore, the potential for a small, non-significant reduction in early-onset sepsis must be weighed against the unknown long-term ecological consequences of altering the neonatal microbiome, an area that requires further investigation.

The conflicting evidence on neonatal outcomes mirrors the findings of other systematic reviews and major guidelines [18,19], which have struggled to formulate unanimous recommendations. For instance, a Cochrane review focusing on term PROM found a reduction in maternal infection but no clear evidence of benefit for the baby, a conclusion that resonates with the findings of Nabhan et al. [14] and Passos et al. [12] in this review. Similarly, the guidelines from the American College of Obstetricians and Gynecologists (ACOG) [20] have historically been more cautious regarding routine antibiotic use in term PROM compared to their strong recommendations for its use in preterm PROM. This caution is rooted in the recognition that the risk-benefit calculus is different at term, where the latency period is typically shorter and the fetal immune system is more mature. Our finding of mixed neonatal outcomes reinforces this cautious stance and suggests that the routine use of antibiotics for the sole purpose of preventing neonatal sepsis in term PROM is not supported by consistent, high-quality evidence.

A critical factor that appears to modulate the effectiveness of antibiotic prophylaxis is the timing of its administration. The review included two studies that directly or indirectly addressed this issue, and they presented conflicting insights. Barišić et al. [13] strongly associated administration within six hours of PROM with improved maternal and neonatal outcomes compared to later administration. This supports the intuitive clinical practice of early intervention to prevent colonization and infection. In contrast, the well-designed study by Dan et al. [11], which used propensity-score matching to control for confounding, found no difference in outcomes whether antibiotics were given within or after 6-12 hours. This null finding challenges the necessity of a strict, narrow window for administration in all cases. This discrepancy could be explained by differences in study populations, definitions of "early" administration, or overall management protocols. It is plausible that in settings with a higher baseline risk of infection or longer average latency periods, the timing of antibiotics is more critical. Conversely, in settings with more active management of labor, the impact of timing may be diminished. This ambiguity highlights an important area for future research, specifically the need for a large, randomized trial designed explicitly to compare different timing strategies for antibiotic administration in term PROM.

When interpreting the collective evidence, the methodological quality of the included studies must be considered. The overall risk of bias was low for most studies, which strengthens confidence in their findings. The large, placebo-controlled RCT by Nabhan et al. [14] and the robust propensity-score matched analysis by Dan et al. [11], both judged as low risk of bias, provide particularly compelling evidence for the null effect of antibiotics on several key outcomes. In contrast, some of the studies showing positive effects, such as the one by Ovalle et al. [15], were judged to have a high risk of bias, potentially overestimating the intervention's benefit. The inclusion of both RCTs and observational studies provides a comprehensive view, but the most reliable conclusions are often drawn from the higher-quality evidence. Therefore, the null findings from the more rigorous studies [11,14] should be given considerable weight when formulating an overall interpretation, suggesting that any benefits of routine antibiotic prophylaxis in term PROM are likely to be modest at best and may not be universal.

The synthesis of these findings suggests that a blanket policy of antibiotic prophylaxis for all women with term PROM is unlikely to be the most prudent approach. Instead, a more targeted strategy may be warranted. Antibiotics may be most beneficial for a subset of women at higher risk of infectious morbidity, such as those with prolonged rupture of membranes, those with known Group B Streptococcus colonization, or those in whom a significant delay in delivery is expected. This risk-based approach is more aligned with the principles of antimicrobial stewardship, a critical consideration in an era of rising antimicrobial resistance. Indiscriminate antibiotic use contributes to resistance and poses potential risks to the neonatal microbiome without a clear, consistent benefit for the majority of mothers and neonates. Therefore, clinical judgment should be exercised to identify women who are most likely to benefit from intervention, rather than applying a universal protocol.

Limitations

This systematic review has several limitations. First, the number of included studies is relatively small, and their methodological heterogeneity, particularly in terms of antibiotic regimens, comparators, and definitions of outcomes, precluded a meta-analysis, limiting the ability to provide a pooled quantitative estimate of effect. Second, the included studies span a considerable time period (1998-2024), during which standard obstetric practices, such as guidelines for Group B Streptococcus prophylaxis and active management of labor, have evolved, which may affect the generalizability and applicability of older studies to current clinical contexts. Third, the assessment of neonatal outcomes was largely limited to early-onset infections; the long-term consequences of antibiotic exposure on the neonatal microbiome and later childhood health outcomes were not evaluated in the included studies and remain an important evidence gap. Finally, while the overall risk of bias was low, some studies, particularly older RCTs, had methodological shortcomings that could influence their results.

## Conclusions

The evidence regarding antibiotic prophylaxis in term PROM is mixed and context-dependent. While certain studies demonstrate a clear benefit in reducing maternal infections, others, including some of the most methodologically robust, show no significant advantage for either maternal or neonatal outcomes. The effect on neonatal sepsis remains particularly uncertain. The timing of administration is a key variable, but the optimal window is not clearly defined by the current evidence. Therefore, the decision to use antibiotic prophylaxis in term PROM should not be routine but should be individualized, considering the patient's specific risk factors for infection and the expected management of labor. Future research should prioritize large, high-quality randomized trials to definitively address the utility of a targeted, risk-based approach versus universal prophylaxis and to further elucidate the impact of timing on clinical outcomes.

## Appendices

Appendix A 

**Table 5:** Search strings for all databases

Database	Search String
PubMed / MEDLINE	("Premature Rupture of Membranes"[MeSH] OR "term PROM" OR "term premature rupture of membranes" OR "prelabor rupture of membranes") AND ("Anti-Bacterial Agents"[MeSH] OR antibiotic* OR prophylaxis OR antimicrobial*) AND ("Maternal Outcome"[MeSH] OR "Neonatal Outcome"[MeSH] OR chorioamnionitis OR endometritis OR "neonatal sepsis")
Web of Science	TS = (("term premature rupture of membranes" OR "term PROM" OR "prelabor rupture of membranes") AND (antibiotic* OR antimicrobial* OR prophylaxis) AND (maternal outcome* OR neonatal outcome* OR chorioamnionitis OR endometritis OR neonatal sepsis))
Embase	('premature rupture of membranes'/exp OR 'term prom' OR 'term premature rupture of membranes' OR 'prelabor rupture of membranes') AND ('antibiotic agent'/exp OR antibiotic* OR antimicrobial* OR prophylaxis) AND ('maternal outcome'/exp OR 'neonatal outcome'/exp OR chorioamnionitis OR endometritis OR neonatal sepsis)
ClinicalTrials.gov	Condition or disease: Premature Rupture of Membranes AND Other terms: term PROM OR antibiotic prophylaxis OR antibiotics AND Outcomes: maternal infection OR neonatal sepsis
